# Seven hundred years after Mondino de Luzzi (1275–1326): method and observation in medieval neuroanatomy

**DOI:** 10.1055/s-0046-1829018

**Published:** 2026-10-02

**Authors:** Veaceslav Merlo Solopa, Sara Rota Conti, Michele Augusto Riva

**Affiliations:** 1 University of Milano-Bicocca, School of Medicine and Surgery, Monza, Italy.; 2 Fondazione IRCCS San Gerardo dei Tintori, Department of Occupational Health, Monza, Italy.

**Keywords:** Mondino de Luzzi, Anatomy, Dissection, Cerebral Ventricles, Knowledge, History of Medicine, History, Medieval

## Abstract

The present paper reexamines the contribution of Mondino de Luzzi (1275–1326) beyond his well-known association with the reintroduction of human dissection in medieval medicine. Focusing on ventricular theory, it explores how Mondino's teaching brought traditional descriptions of the brain into closer contact with the observation of the human body through public dissection. Although his work remained firmly rooted in Galenic and Avicennian traditions, the association of cerebral ventricles with identifiable bodily structures indirectly rendered established doctrines less purely speculative and more susceptible to anatomical scrutiny and criticism. From the Renaissance onward, this contributed to a broader reevaluation of inherited anatomical and physiological theories.

## INTRODUCTION

Seven hundred years have passed since the death of Mondino de Luzzi (1275–1326), one of the foundational figures of anatomy in Western medicine. Historians have praised his contributions to the history of medicine, especially for the reintroduction of the study of anatomy through hands-on pedagogical dissections. 1 2 The aim of the current paper is not to provide another biographical account of this author; rather, it is argued that Mondino's contribution extends beyond his well-known role in the revival of anatomical dissection, and it includes a subtle but significant epistemological transformation. While addressing his broader place in the history of neuroanatomy, the present study focuses on the ventricular theory as a case through which the methodological consequences of this shift can be explored.

## VENTRICULAR THEORY BEFORE MONDINO

Before Mondino, neuroanatomical knowledge was largely shaped by authoritative texts and conceptual models rather than by systematic anatomical investigation. While multiple traditions contributed to medieval understandings of the brain, ventricular theory emerged as the dominant explanatory framework for mental faculties. The description of the cavities of the brain is usually attributed to Aristotle (384–322 BCE); illustrious figures such as Galen (c. 129–216 CE) and Avicenna (980–1037) subsequently made additions and reinterpretations, helping to shape a coherent framework of the workings of the mind. 3 4 Galen provided the initial idea that the ventricles were linked to cognition, but it was Avicenna who assigned specific faculties to three supposedly-distinct ventricles.

According to late medieval ventricular theory, mental faculties were mediated by the *spiritus animalis* , which circulated and was modified within the ventricles, while the surrounding brain tissue served a primarily-structural role, as illustrated in Figure 1 . Fluid within the ventricles was considered particularly suitable for the rapid inner mechanisms of the mind, precisely because fluid was thought to move, transform, and mediate action. In contrast, solid brain tissue was viewed as cold and static. The theory further posited that the anterior ventricle was responsible for integration and imagination, the middle ventricle, for reasoning, and the posterior ventricle, for memory. Although conceptually cohesive, the ventricular theory remained sufficiently vague to enable reinterpretation when anomalies were observed. 3

**Source:Figure 1:**
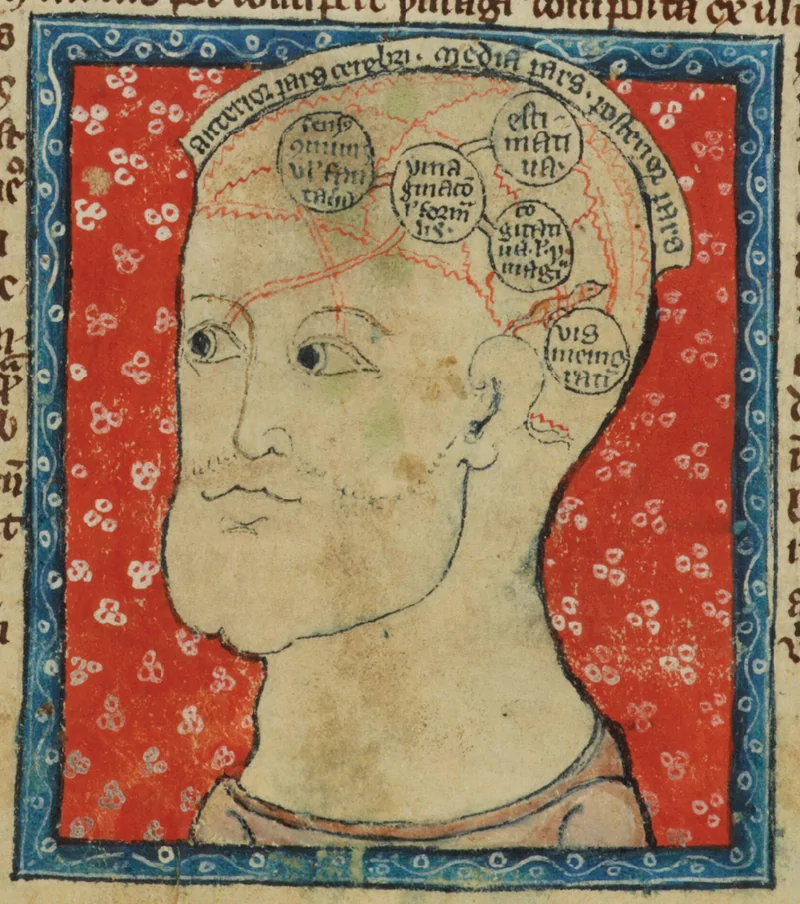
Note: Image reproduced under a Creative Commons Attribution–NonCommercial 3.0 Unported License (CC BY-NC 3.0). Cambridge Digital Library: https://cudl.lib.cam.ac.uk/view/MS-GG-00001-00001/988 (accessed June 1, 2026) Illustration from a 1307 trilingual compendium depicting the 5 faculties of the brain: perception, imagination, estimation, cogitation, and memory. The image accompanies the short treatise *Qualiter caput hominis situatur* (“How the Human Head Is Structured”) and appears in a collection of texts preserved in Cambridge University Library (MS Gg.1.1, fol. 490v).

## THE ANATOMICAL RECONFIGURATION OF VENTRICULAR THEORY

In the Middle Ages, the bodies of the deceased were destined for resurrection and were therefore to be treated with reverence. Human dissections remained relatively uncommon in much of medieval Europe and were shaped by religious, social, and juridical considerations. However, the city Bologna constituted a context more favorable to the practice of human dissection, due to the presence of an active judicial system, characterized by relatively-frequent executions, together with the close interaction between jurists and physicians within the university. 1 5

It was in this context that Mondino de Luzzi emerged as a prominent physician and professor. His father owned a pharmacy and his uncle was a medical professor at Università di Bologna. 6 He therefore grew up in close contact with the medical remedies and practices of his time, within an environment that likely encouraged his practical approach to medicine. 1 Mondino's knowledge culminated in the writing of *Anathomia* in 1316. In this work, he describes the procedure to dissect the brain, a particularly-challenging organ due to its rapid liquefaction upon exposure to air. Mondino describes the dura mater and pia mater and proceeds to distinguish the anterior brain, the cerebrum, from the posterior brain, the cerebellum. 1 7

Given the central importance of ventricular theory, Mondino describes the ventricles in detail. In his account, the three functional areas were ascribable to four ventricles ( Figure 2 ). The anterior ventricle was indeed subdivided into left and right components corresponding to what are now known as the lateral ventricles. Fantasy, common sense, and imagination were attributed respectively to the anterior, middle, and posterior parts of this anterior ventricle. The middle ventricle served as a passage to the posterior ventricle and was responsible for meditation and reasoning faculties. The posterior ventricle served the function of memory. 2 7

**Source:Figure 2:**
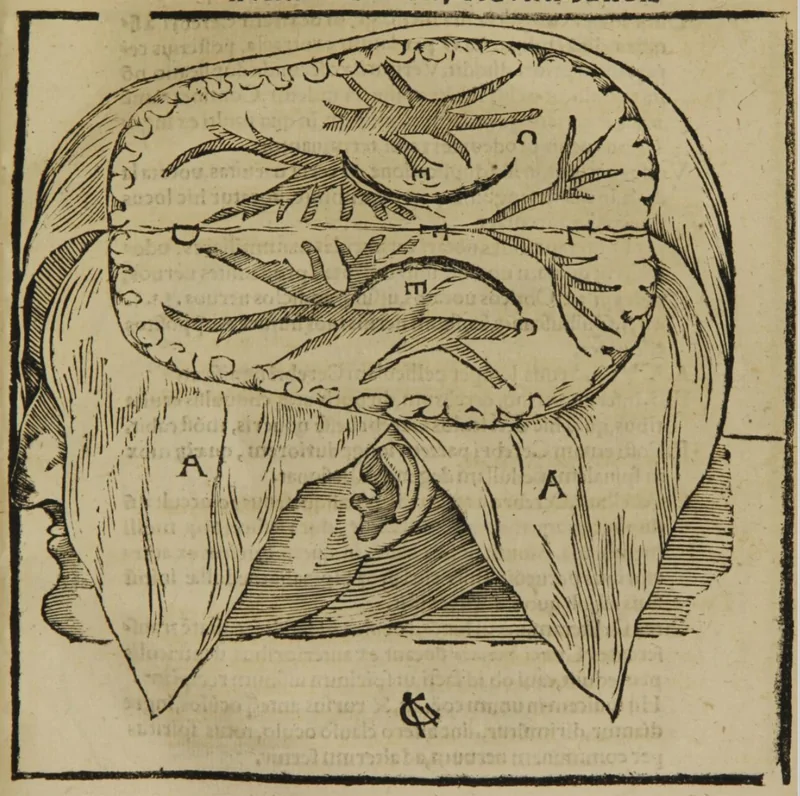
Note: Image from the collections of Bibliothèque Interuniversitaire de Santé (Paris), made available under the Etalab Open License. Internet Archive, Medical Heritage Library edition: https://archive.org/details/BIUSante_05544x01/page/n141/mode/2up (accessed June 1, 2026) Sagittal section of the human brain within the skull, reproduced from the 1541 illustrated edition of *Anathomia* by Mondino de Luzzi. The woodcut reflects late medieval anatomical conceptions of the cerebral ventricles and their presumed functions. Published in *Anatomia Mundini, ad vetustissimorum erundemque aliquot manu scriptorum codicum fidem collata, justoque suo ordini restituta* , edited by Jonas Dryander (Marburg: Christian Egenolff, 1541).

Mondino's description closely resembles those of Galen and Avicenna; however, an important distinction emerges: Galen's ventricular theory was mainly physiological and not intended as a precise anatomical map. Avicenna later developed this idea by assigning specific mental faculties to the three ventricles, treating them as functional rather than strictly-anatomical spaces. 4 Mondino's main contribution was to describe these spaces in anatomical terms. By associating mental functions with identifiable cerebral structures, he indirectly contributed to rendering the ventricular theory less purely speculative and, in the centuries that followed, more susceptible to anatomical scrutiny and criticism.

In conclusion, Mondino's contribution to anatomy is often associated with his role in the introduction of human dissection as a teaching practice. Mondino dissected publicly in front of his students, following a sequence informed by philosophical assumptions while remaining constrained by practical considerations such as time, decomposition, and access to anatomical structures. 1 5 Within this ordered sequence, Mondino consistently placed the dissection of the brain at the end of the procedure. This choice conferred a distinctive status on the brain in comparison with other organs. By reserving it for the final stage of dissection, Mondino treated the brain as a structure requiring particular care and attention, in keeping with its central role in the organization of mental faculties. Notably, this practice persists in modern cadaveric dissections, in which the brain is often examined last.

Although Mondino largely remained within the Galenic tradition, his significance lies not only in his anatomical descriptions, but also in the educational framework he helped consolidate. The collective practice of public dissection brought written anatomical doctrines into closer contact with direct observation of the human body, reducing individual variation and making discrepancies between traditional models and actual anatomy more apparent. Over the following centuries, particularly from the Renaissance onward, this contributed to an environment in which inherited anatomical and physiological theories could be more readily scrutinized and revised.
